# Effects of Perfluorooctane Sulfonate on Cerebellar Cells via Inhibition of Type 2 Iodothyronine Deiodinase Activity

**DOI:** 10.3390/ijms241612765

**Published:** 2023-08-14

**Authors:** Yuki Fujiwara, Yuhei Miyasaka, Ayane Ninomiya, Wataru Miyazaki, Toshiharu Iwasaki, Winda Ariyani, Izuki Amano, Noriyuki Koibuchi

**Affiliations:** 1 Department of Integrative Physiology, Gunma University Graduate School of Medicine, Maebashi 371-8511, Japan; y-fujiwara@gunma-u.ac.jp (Y.F.); m1820602@gunma-u.ac.jp (A.N.); winda@gunma-u.ac.jp (W.A.); iamano-lj@umin.ac.jp (I.A.); 2 Gunma University Heavy Ion Medical Center, Maebashi 371-8511, Japan; y.miyasaka@gunma-u.ac.jp; 3 Department of Bioscience and Laboratory Medicine, Hirosaki University Graduate School of Health Science, Hirosaki 036-8564, Japan; miya@hirosaki-u.ac.jp; 4 Horie Hospital, Ota 373-8601, Japan; tiwasaki10@hotmail.co.jp

**Keywords:** per- and polyfluoroalkyl substances, perfluorooctane sulfonate, Purkinje cell, thyroid hormone, thyroid hormone receptor, cerebellar development, type 2 iodothyronine deiodinase

## Abstract

Perfluorooctane sulfonate (PFOS) has been used in a wide variety of industrial and commercial products. The adverse effects of PFOS on the developing brain are becoming of a great concern. However, the molecular mechanisms of PFOS on brain development have not yet been clarified. We investigated the effect of early-life exposure to PFOS on brain development and the mechanism involved. We investigated the change in thyroid hormone (TH)-induced dendrite arborization of Purkinje cells in the primary culture of newborn rat cerebellum. We further examined the mechanism of PFOS on TH signaling by reporter gene assay, quantitative RT-PCR, and type 2 iodothyronine deiodinase (D2) assay. As low as 10^−7^ M PFOS suppressed thyroxine (T_4_)-, but not triiodothyronine (T_3_)-induced dendrite arborization of Purkinje cells. Reporter gene assay showed that PFOS did not affect TRα1- and TRβ1-mediated transcription in CV-1 cells. RT-PCR showed that PFOS suppressed D2 mRNA expression in the absence of T_4_ in primary cerebellar cells. D2 activity was also suppressed by PFOS in C6 glioma-derived cells. These results indicate that early-life exposure of PFOS disrupts TH-mediated cerebellar development possibly through the disruption of D2 activity and/or mRNA expression, which may cause cerebellar dysfunction.

## 1. Introduction

Perfluorooctane sulfonate (PFOS) is a member of bioaccumulative per- and polyfluoroalkyl substances (PFAS). Because of its amphipathic nature, PFOS was used as a surfactant in fire-fighting foams, a treatment of paper, clothes, carpets, and leather products, metal plating, and coating additives. [1,2,3,4]. On the other hand, PFOS behaves like persistent organic pollutants (POPs) due to the chemical stability against hydrolysis, photolysis, acidic and basic attack, oxidizing and reducing agents, and biodegradation [5]. Nowadays, PFOS has been known as an environmental chemical to accumulate in the soil, water, air, and biota [6], meaning humans are easily exposed to PFOS in everyday life. As a consequence, its toxicity to human health has been widely reported, including hepatoxicity [7,8], reproductive toxicity [9], endocrine disruption [10], and carcinogenicity [11], and thus is considered a worldwide problem.

In particular, PFOS can be detected in maternal serum from the placenta [12], umbilical cord blood [13], and breast milk [14], and there is concern that its transfer to the brain may have adverse developmental effects. Indeed, PFOS concentration in the cord serum has negative associations with birth weight and head circumference in humans [15]. Some epidemiological studies showed a negative correlation between PFOS concentration in the maternal serum and intelligence or motor performance in children [16,17]. In a mouse model, we previously found that the lactational PFOS exposure disrupts learning and memory in adult male offspring [18]. We also found impairments in motor coordination and motor learning in adult male offspring following the lactational PFOS exposure [19]. As this suggested that the lactational PFOS exposure causes the aberrant development of the cerebellar function, we observed the attenuation of pre- and postsynaptic plasticity at parallel fiber Purkinje cell synapses [19]. In other animal studies, neonatal exposure to PFOS altered the expression of proteins critical for neuronal growth and synaptogenesis in mice [20] and caused neurobehavioral defects [21]. However, the mechanism of PFOS neurotoxicity to synaptogenesis and synaptic plasticity has remains to be elucidated. 

Purkinje cells, the sole output neurons from the cerebellar cortex, have crucial neuronal functions [22]. The development of Purkinje cells is largely regulated by the thyroid hormone (TH) [23]. Previous studies indicate that hypothyroidism or overexpression of mutant TH receptor (TR) in the cerebellum induces changes in the arborization of dendritic trees and synapse formation of Purkinje cells [24,25,26]. TH exerts its effects on cerebellar development by binding to TRs which are abundantly expressed in the cerebellum. In rodents, TH, particularly thyroxine (T_4_), crosses the blood–brain barrier mainly through organic anion transporting polypeptide (OATP) 1C1 and is taken up by astrocytes. In the cerebellar astrocytes, 5′-deodination of T_4_ is converted to triiodothyronine (T_3_) by type 2 iodothyronine deiodinase (D2). Then, T_3_ is transferred to neurons including Purkinje cells or oligodendrocytes through monocarboxylate transporter (MCT) 8 and binds to nuclear TR to regulate transcription of its target genes [27]. As a result, perinatal hypothyroidism suppresses TH-responsive genes in the Purkinje cell, such as the retinoic acid receptor-related orphan receptor (ROR)alpha, inositol 1, 4, 5-triphosphate receptor type 1 (IP3R1), and sarco/endoplasmic reticulum Ca^2+^-ATPase 2 (SERCA2), resulting in aberrant Purkinje cell development [28].

Previous studies indicate that the TH-mediated dendritic growth of Purkinje cells was modified by several environmental chemicals such as polychlorinated biphenyls (PCBs) and polybrominated diphenyl ethers (PBDEs) [29,30,31]. We previously reported that such environmental chemicals altered the dendritic arborization of Purkinje cells by suppressing the TR-mediated transcription [30,31,32,33,34]; however, it has not been directly clarified whether PFOS affects TH-induced cerebellar development through suppressing TR action or D2 activity. In the present study, we investigated the PFOS effects on dendritogenesis of Purkinje cells in primary cerebellar culture and its possible mechanisms involved.

## 2. Results

### 2.1. PFOS Disrupts TH-Induced Dendrite Arborization of Cerebellar Purkinje Cells

**Figure 1:**
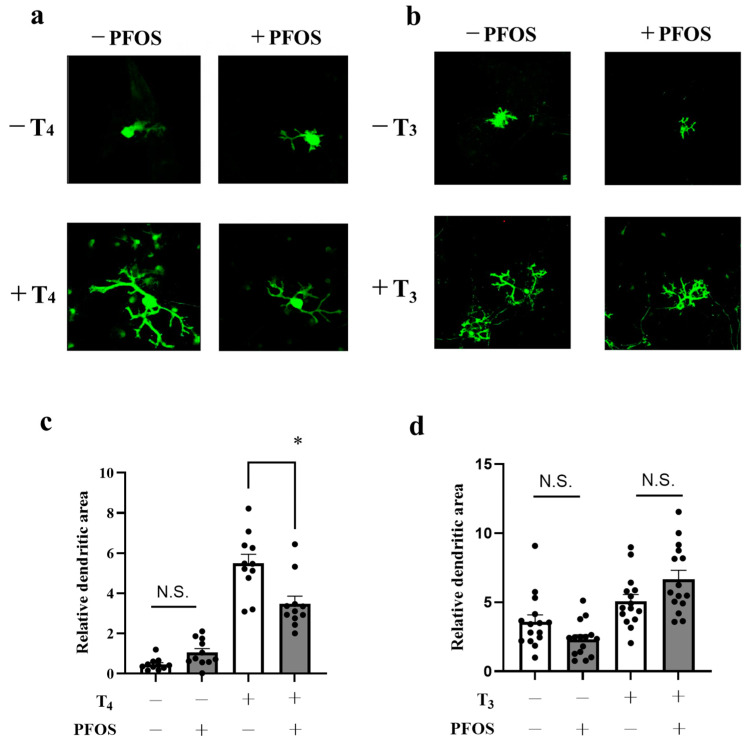
Effect of perfluorooctane sulfonate (PFOS) on dendrite arborization of Purkinje cells. (**a**) Representative photomicrographs showing the effect of PFOS on Purkinje cell morphology (17 days in culture). PFOS (10^−7^ M) was added to the culture in the absence or presence of thyroxine (T_4_) (10^−8^ M), and immunocytochemistry was performed using anti-calbindin antibody to visualize Purkinje cells. (**b**) Photomicrographs showing the effect of PFOS on Purkinje cell morphology in the presence of triiodothyronine (T_3_) (17 days in culture). PFOS (10^−7^ M) was added to the culture in the presence of T_3_ (10^−8^ M), and immunocytochemistry was performed using anti-calbindin antibody to visualize Purkinje cells. (**c**) Change in dendritic areas of Purkinje cells after PFOS addition. In (**c**), data are expressed as mean ± SEM (*n* = 15 determinations) from one experiment and represent at least three independent experiments. (**d**) Change in dendritic areas of Purkinje cells after PFOS addition in the presence of T_3_. In (**d**), data are expressed as mean ± SEM (*n* = 11 determinations) from one experiment and represent at least three independent experiments. * *p* < 0.05, N.S. = no significant.

We studied the effect of PFOS on T_4_-induced dendrite arborization of cerebellar Purkinje cells in primary culture. Of note, since the primary culture contains all cell types of the cerebellum and has a condition similar to the native circumstance, we used T_4_ as a stimulant. In the cerebellum, under physiological conditions, T_4_ is first taken up by astrocytes, converted to T_3_, which has a higher affinity for thyroid receptors than T_4_ and transported to other cerebellar cells where it induces various physiological effects. Seventeen days after the onset of culture, cells were fixed and immunostained with an anti-calbindin antibody to visualize Purkinje cells. The addition of PFOS (10^−7^ M) to the culture together with T_4_ (10^−8^ M) inhibited the TH-induced development of Purkinje cell dendrites. The dendrites showed very poor growth, and the secondary branches were especially small (Figure 1a); the area of these Purkinje cell dendrites was significantly reduced by PFOS (Figure 1a,c). In the absence of T_4_, PFOS-treated Purkinje cells showed almost complete absence of dendrites (Figure 1a). Quantitative analysis showed an approximately 40% decrease in the dendritic area by PFOS treatment (Figure 1b). These data indicate that PFOS may disrupt cerebellar development by inhibiting TH-mediated Purkinje cell development. To our surprise, PFOS did not affect the dendrite arborization of Purkinje cells in the presence of 10^−8^ M T_3_ (Figure 1b,d). No particular morphological changes were noted by microscopic examination (Figure 1b). Quantitative analysis showed no changes in dendritic areas by PFOS treatment with T_3_ (Figure 1d). Thus, PFOS showed differential effects on T_4_- or T_3_-induced Purkinje cell dendritogenesis.

### 2.2. TRα1- and TRβ1-Mediated Transcriptions Were Not Affected by PFOS in CV-1 Cells

**Figure 2:**
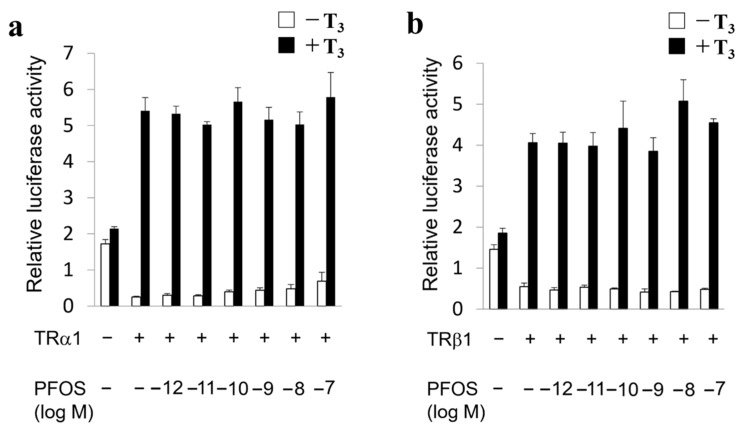
Thyroid hormone receptor (TR)-mediated transcriptions were not altered by perfluorooctane sulfonate (PFOS). Plasmids encoding TRα1 ((**a**) 10 ng) or TRβ1 ((**b**) 10 ng) were co-transfected with chick lysozyme-thyroid hormone response element (100 ng) into CV-1 cells. The cells were incubated with or without 10^−7^ M triiodothyronine (T_3_) and the indicated amounts of PFOS. The total amount of DNA in each well was balanced by adding the vector pcDNA3. Data are mean ± SEM of experiments performed in triplicate.

To examine the mechanisms of the PFOS toxicity disrupting cerebellar development, we examined the effect of various concentrations of PFOS on TR-mediated transcription in fibroblast-derived CV-1 cells using the transient transfection-based reporter gene assay. Representative results are shown in Figure 2. Even 10^−7^ M of PFOS did not affect the TRa1- or TRb1-mediated transcription (Figure 2). In addition, PFOS did not alter GR- or ERα- mediated transcription (see Supplemental Material, Supplementary Figure S1a,b). These results indicate that transcriptional suppression may not be the reason for the suppression of T_4_-mediated dendrite arborization of Purkinje cells by PFOS.

### 2.3. D2 mRNA Expression Was Suppressed by PFOS

**Figure 3:**
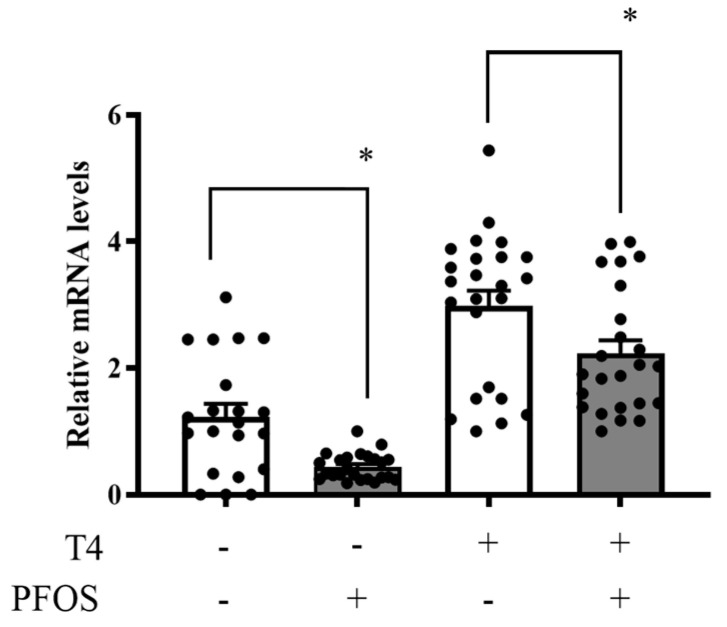
Suppression of type 2 iodothyronine deiodinase (D2) mRNA expression of D2 in primary cerebellar culture. Cerebellar cells in 24-well plates were treated with indicated concentrations of perfluorooctane sulfonate (PFOS) and/or thyroxine (T_4_). This figure shows the D2 mRNA levels normalized to glyceraldehyde 3-phosphate dehydrogenase mRNA levels (mRNA D2/GAPDH) from the same RT reaction for each sample. The results show the ratio of each sample relative to that of the control. Data are presented as mean ± SEM, *n* = 21. The values for the control group were 1. * *p* < 0.05.

Quantitative reverse transcription polymerase chain reaction (RT-PCR) was performed to examine the effect of PFOS on D2 mRNA expression in primary cerebellar cultures. In the absence of T_4_, PFOS suppressed the D2 mRNA expression (Figure 3). T_4_ increased the expression of D2 mRNA compared with that in the control group; however, even in the presence of T_4_, PFOS suppressed D2 mRNA expression (Figure 3).

### 2.4. D2 Activity Was Suppressed by PFOS

**Figure 4:**
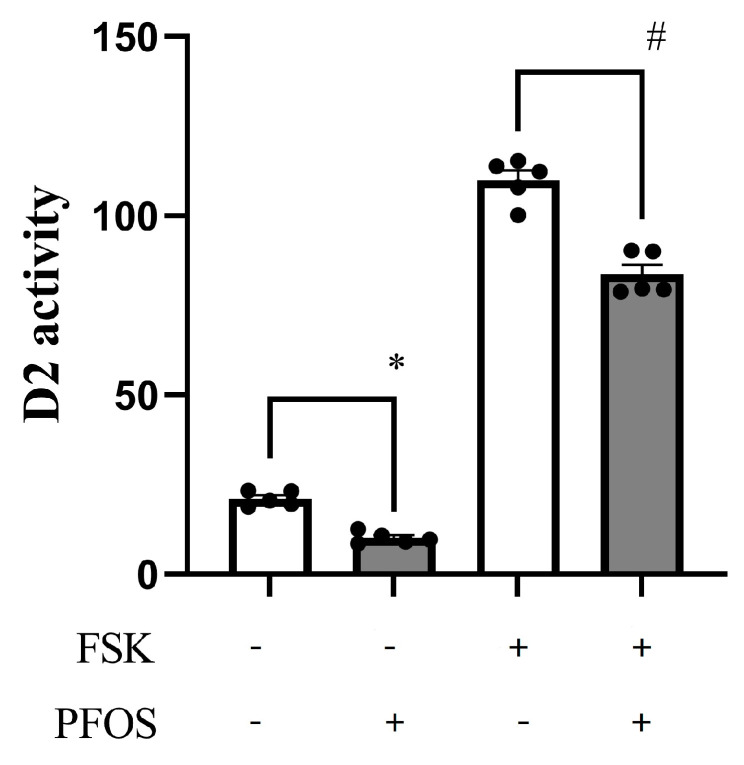
Change in type 2 iodothyronine deiodinase (D2) activity in C6 cells. C6 cells were cultured with indicated concentrations of perfluorooctane sulfonate (PFOS) and/or forskolin (FSK) for 3 h. D2 activity was measured by measuring free ^125^I levels using a γ-counter. Values are presented as means ± SEM of three experiments performed in triplicate (*n* = 5). # *p* < 0.001, * *p* < 0.05.

**Figure 5:**
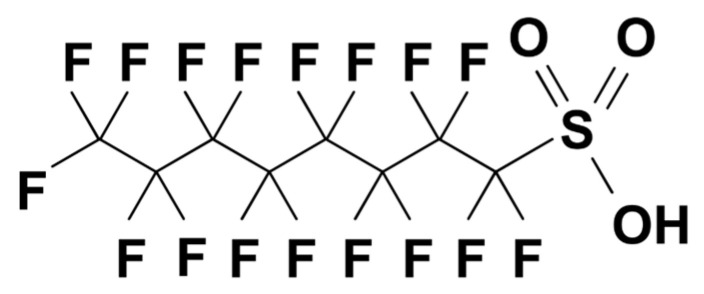
Chemical structure of perfluorooctane sulfonate (PFOS).

To examine the D2 activity in astrocytes, we carried out D2 activity assays using C6 cell extract (Figure 4). In the absence of T_4_, PFOS significantly suppressed D2 activity. To confirm the suppression of D2 activity by PFOS, we added forskolin (FSK) to induce D2 activity in the absence of T_4_. FSK increases intracellular levels of cAMP by activating adenylyl cyclase [35]. D2 is a cAMP-responsive gene and thus the increase in intracellular cAMP activates the transcriptional of the D2 gene [36]. As shown in Figure 5, FSK treatment dramatically increased the D2 activity by five-fold. PFOS significantly suppressed D2 activity. These results indicate that suppression of D2 activity by PFOS may result in the suppression of dendrite arborization of Purkinje cells in primary culture.

## 3. Discussion

In the present study, we demonstrated that as low as 10^−7^ M of PFOS suppressed the T_4_-induced dendrite arborization of Purkinje cells in the primary cerebellar culture, whereas T_3_-induced dendrite arborization was not suppressed. PFOS did not affect the TR-mediated transcription in CV-1 cells. Finally, we showed that PFOS suppressed the D2 activity in C6 rat astrocytoma-derived cells and mRNA expression in the cerebellar primary culture. These results indicate that PFOS suppressed cerebellar development by inhibiting the conversion of T_4_ to T_3_ in the astrocyte.

We initially hypothesized that the suppression of Purkinje cell dendritic arborization may be due to the suppression of TR-mediated transcription because most of our previous studies used several environmental chemicals such as polychlorinated biphenyls (PCBs), polybrominated diethyl ethers (PBDEs), 1,2,5,6,9,10-a hexabromo- cyclododecane (HDCC), and the polybrominated biphenyl mixture, BD-6, revealed that suppression of TR-mediated transcription by these chemicals induced the suppression of dendrite arborization of Purkinje cells [30,31,32,33]. However, primary culture studies have shown that PFOS suppressed T_4_-mediated dendrite arborization of Purkinje cells, but not T_3_-mediated arborization. It was our first experience to observe such a differential effect. Furthermore, our reporter gene assay showed that PFOS did not alter TRα1- or TRβ1-mediated transcription (Figure 3). Since our cerebellar primary culture contains all sets of cerebellar cells, including astrocytes where T_4_ is converted to T_3_, an active form of TH, we presumed that PFOS may affect D2 activity or expression. Thus, we measured D2 mRNA levels by quantitative PCR and found that PFOS suppressed D2 mRNA levels both in the presence and absence of T_4_. However, because D2 activity does not always equal to D2 mRNA levels, we considered it necessary to measure D2 activity. 

To measure the D2 activity, the primary culture contains too little astrocyte. Thus, other systems to study the effect of PFOS on D2 activity need to be applied. We used C6 astrocytoma-derived clonal cells. The nature of C6 cells is similar to those of astrocytes and many previous studies used this cell line as a model system to study astrocyte biology [37,38]. To examine the cell viability, we first carried out a cell proliferation assay and confirmed that PFOS did not affect cell viability (Supplementary Figure S2). However, we observed a decrease in the D2 mRNA level in primary cerebellar culture. These results are in line with a previous study showing a decrease in D2 mRNA in *Perca fluviatilis* [39]. 

To examine the effect of PFOS on D2 activity, we carried out a D2 activity assay. We found that 10^−7^ M PFOS significantly suppressed the D2 activity in C6 cells in the absence or presence of 10^−10^ or 10^−9^ M T_4_. Since D2 expression is upregulated by intracellular cAMP, we stimulated D2 activity by FSK treatment and examined the effect of PFOS. PFOS significantly inhibited FSK-stimulated D2 activity. Taken together with the PFOS effect on D2 mRNA levels, these results indicate that PFOS may at least in part inhibit Purkinje cell dendrite arborization by inhibiting the conversion of T_4_ to T_3_.

Although the mechanisms inhibiting D2 activity have not yet been clarified, PFOS may disrupt the adenylyl cyclase/cAMP response element binding protein (CREB) system to alter the signal transduction pathway. A previous study has shown that perinatal PFOS exposure induced an alteration of several calcium-dependent signaling molecules, including CREB in the hippocampus [40]. Such alteration may have disrupted D2 synthesis and activation and inhibited T_4_ conversion to reduce the local T_3_ level in the cerebellum. However, additional studies may be required to confirm such a possibility.

In addition to the effect of PFOS to disrupt TH action by inhibiting D2 activity, PFOS may affect thyroid function. Berg et al. reported that PFOS was positively associated with TSH levels in pregnant women [41]. Kato et al. reported that Maternal PFOS levels were inversely correlated with maternal serum TSH and positively associated with infant serum TSH [42]. As PFOS may act through multiple pathways such as to disrupt D2 activity and TSH secretion, PFOS may disrupt TH-induced brain development, including the cerebellum. We previously reported that lactational PFOS exposure caused the functional development of the cerebellum, underlined as an impairment in motor coordination and synaptic plasticity at parallel fiber Purkinje cell synapses [19]. Although we did not examine the involvement of the TH system in the previous study, it could be likely that the Purkinje cell function is modified by the reduced D2 activity by PFOS. Further studies may be required to confirm such a possibility.

## 4. Materials and Methods

### 4.1. Chemicals

T_3_, T_4_, dexamethasone, and β-estradiol (E_2_) were purchased from Sigma-Aldrich (St. Louis, MO, USA). T_3_ and T_4_ were dissolved in dimethyl sulfoxide (DMSO) or 0.01 N NaOH at a concentration of 100 mM. PFOS (purity > 98%) was purchased from Fluka Chemicals (Buchs, Switzerland). The chemical structure of PFOS is shown in Figure 5. PFOS was dissolved in methanol or distilled water containing 0.5% Tween 20 (Sigma-Aldrich) at 100 mM. The culture medium was diluted to the indicated concentrations with stock solution (10^−3^ M) immediately before use. Repeated freeze–thaw cycles were avoided.

### 4.2. Animals

Pregnant Wistar rats were purchased from Japan SLC Inc. (Hamamatsu, Japan). The rats were housed in a temperature- and humidity-controlled room (22–24 °C, 30–60% humidity), and were maintained under a 12 h light–dark cycle (lights on from 7:00 a.m. to 19:00 p.m.). Food and water were provided ad libitum. The animal experimentation protocol used in this study was approved by the Animal Care and Experimentation Committee of Gunma University, Showa Campus. All efforts were made to minimize the number of animals used and their suffering.

### 4.3. Plasmids

The expression vectors for TRβ1, GR, and ER were used as previously described [32,43]. Luciferase (LUC) reporter constructs containing the thyroid hormone response element and chick lysozyme-thymidine kinase-LUC were developed as described previously [44]. A mouse mammary tumor virus (MMTV) promoter containing a glucocorticoid response element fused to the LUC promoter (MMTV-LUC) and 2x estrogen response element (ERE) containing a luciferase reporter (2xERE-LUC) were developed as described previously [44,45].

### 4.4. Clonal Cell Culture

Rat C6 astrocytoma-derived clonal cells were donated by Dr. F. Okajima of Gunma University [37,46]. CV-1 and C6 cells were maintained in Dulbecco’s modified Eagle’s medium (Thermo Fisher Scientific, Waltham, MA, USA), supplemented with 10% small lipophilic hormone-deprived fetal bovine serum (FBS) and antibiotics (100 U/mL penicillin, 100 mg/mL streptomycin) at 37 °C, 5% CO_2_. C6 cells were cultured in medium containing stripped FBS for 6 h before each experiment.

### 4.5. Primary Cerebellar Culture

Cerebellar cultures were prepared as described previously [29]. Briefly, newborn rats were decapitated under ketamine (100 mg/kg)-xylazine (10 mg/kg) anesthesia on postnatal day (P)2. The cerebella from P2 newborn rats were dissected and digested with 0.2 units/mL of papain (Worthington, Lakewood, NJ, USA) in phosphate-buffered saline (PBS) containing 0.2 mg/mL l-cysteine, 0.2 mg/mL bovine serum albumin (BSA) (Thermo Fisher Scientific), 5 mg/mL glucose, and 0.02 mg/mL DNase I (Sigma, 400–600 units/mg) for 25 min at 36.5 °C. Dissociated cells were suspended in a serum-free medium without TH and plated at a density of 2.5 × 10^5^ cells/0.2 mL in wells of chamber slides (8-mm-diameter wells, Nalge Nunc International, Rochester, NY, USA), pre-coated with 0.1 mg/mL poly-l-lysine (Sigma-Aldrich). One day after plating, T_3_, T_4_, and/or PFOS were added to the culture media, half of the medium was replaced with fresh medium every 2 days, and mixed cerebellar cells were cultivated in a 5% CO_2_ incubator for 17 days. The effects of DMSO were excluded using control and experimental media at a final concentration of 0.01%, avoiding repeated freezing and thawing. PFOS addition did not alter the pH of the culture medium.

### 4.6. Analysis of Purkinje Cell Morphology via Immunocytochemistry for Calbindin

Immunocytochemistry of cultured cells was performed as described previously [29]. Briefly, Purkinje cells were immunostained with mouse monoclonal anti-calbindin-28 K antibody (1:1000; McAB 300, Swant, Bellinzona, Switzerland) and fluorescein isothiocyanate-labeled donkey anti-mouse IgG antibody (1:200; Molecular Probes, Eugene, OR, USA), and observed under a laser confocal scanning microscope (FV1000D spectral type inverted microscope IX81, Olympus, Tokyo, Japan). Dendrite arborization, the total area covered by the dendritic tree on randomly selected Purkinje cells in each experiment, was quantified by tracing the outline of the cell and dendritic branches and computing the area using ImageJ software 2.11 (NIH, Bethesda, MD, USA). Ten randomly selected Purkinje cells were used in each culture plate because of the limitations of photobleaching associated with the use of laser confocal microscopy. Data shown represent the mean ± standard error of the mean (SEM), and results from one experiment are shown graphically. More than three independent experiments were performed, and the results were consistent for each experiment. The relative dendritic areas of the Purkinje cells are shown.

### 4.7. Cell Proliferation Assay

Cells in 96-well plates were incubated with or without the PFOS for 24 h. Cultured cells were counted using the CellTiter 96 AQueous One Solution Cell Proliferation Assay Kit (Promega, WI, USA) according to the manufacturer’s instructions.

### 4.8. Transient Transfection-Based Reporter Gene Assay

Cultured C6 cells were plated in 24-well plates 48 h before transfection using the calcium-phosphate coprecipitation method [32]. The cytomegalovirus-β-galactosidase plasmid was used as an internal control. Sixteen to twenty-four h after transfection, the wells were refilled with fresh medium containing the indicated concentrations of the ligand and/or PFOS for twenty-four h. Cells were harvested to measure LUC activity as described previously [32]. The total amount of DNA per well was balanced by adding the pcDNA3 plasmid (Thermo Fisher Scientific). LUC activity was normalized to that of β-galactosidase and then calculated as the relative LUC activity. All transfection experiments were repeated at least thrice. Data shown represent mean ± SEM of one experiment.

### 4.9. Quantitative Real-Time PCR

Total RNA was extracted from C6 cells in 6-well plates using RNeasy kits (QIAGEN, Hilden, Germany). First-strand complementary DNA (cDNA) was prepared from total RNA using a PrimeScript RT Reagent Kit (TAKARA, Kyoto, Japan), and quantitative real-time PCR was performed in a StepOne thermal cycler (Thermo Fisher Scientific) using THUNDERBIRD SYBER qPCR Mix (TOYOBO, Osaka, Japan) for 40 cycles as follows: denaturation at 95 °C for 30 s followed by annealing/extension at 95 °C for 30 s. Data were analyzed using the ΔΔCt method. Glyceraldehyde 3-phosphate dehydrogenase (GAPDH) was used for normalization. The following primer sequences were used in this study: *Dio2* (forward, 5′-AAGCGTCGGAAGCGGGTCAAC-3′; reverse, 5′-GCCAAGCCAACAATCAAGGTG-3′); *Gapdh* (forward, 5′-GTGACAAAGTGGACATTGTTGCC-3′; reverse, 5′-GATGATGACCCTTTTGGCCCC-3′). All experiments were repeated thrice to confirm the consistency of the results.

### 4.10. Measurement of D2 Activity

Although the primary cerebellar culture used in the present study contained astrocytes, which mainly express D2, D2 activity was below the detectable limit, probably due to the limited astrocyte population in the culture; therefore, we used astrocytoma-derived C6 cells for the D2 activity assay. D2 activity was measured as previously described [47], with minor modifications [48]. Briefly, C6 cells in each well were washed twice with PBS, scraped, and transferred to 1.5-mL ice-cold assay buffer (100 mM potassium phosphate [pH 7.0] containing 1 mM EDTA and 20 mM dithiothreitol). After centrifugation at 3000 rpm for 10 min at 4 °C, the resulting precipitates were sonicated in 100 μL of the assay buffer per tube and were incubated in a total volume of 50 μL with 2 nm or the indicated amount of [^125^I]T_4_ or [^125^I]rT_3_, which were purified via LH-20 column chromatography on the day of the experiment in the presence or absence of 1 mm 6-propyl-2-thiouracil (PTU) or in the presence of 1 mm iopanoic acid for indicated periods at indicated temperatures in duplicate. After characterization of deiodination activity in C6 cells, the sonicates were routinely incubated with 2 nm [^125^I] T_4_ in the presence of 1 mM PTU at 37 °C for 1 h. The reaction was terminated by adding 100 μL ice-cold 2% BSA and 800 μL ice-cold 10% trichloroacetic acid. After centrifugation at 3000 rpm for 10 min at 4 °C, the supernatant was applied onto a small column packed with AG 50W-X2 resin (bed vol = 1 mL) and then eluted with 2 mL of 10% glacial acetic acid (column method). Separated ^125^I was counted with a γ-counter. Nonenzymatic deiodination was corrected by subtracting I^−^ released in control tubes without cell sonicates. The protein concentration was determined by Bradford’s method using BSA as the standard. The deiodination activity was calculated as femtomoles of I^−^ released/mg protein after multiplying by a factor of two to correct random labeling at the equivalent 3′ and 5′ positions.

### 4.11. Statistical Data Analysis

Data were analyzed using one-way analysis of variance. A post hoc comparison was performed using Bonferroni’s test. All data in the text and figures are expressed as the mean and SEM. Statistical significance was set at *p* < 0.05.

## 5. Conclusions

In conclusion, PFOS may affect T_4_-induced dendrite arborization of Purkinje cells, at least in part through the suppression of D2 activity. Although many in vitro screening systems of environmental chemicals apply the transcription assay, such a single method may not be sufficient for the screening of thyroid hormone system-disrupting chemicals. Thus, we examined its toxicity in vitro by applying multiple approaches. Our study may contribute to clarifying the molecular mechanism of PFOS toxicity in the developing cerebellum.

## Data Availability

The data presented in this study are available on request from the corresponding author.

## Supplementary Materials

The following supporting information can be downloaded at: https://www.mdpi.com/article/10.3390/ijms241612765/s1.
