# Supplementary material for: Effects of Perfluorooctane Sulfonate on Cerebellar Cells via Inhibition of Type 2 Iodothyronine Deiodinase Activity
Source: Int J Mol Sci. 2023 Aug 14;24(16):12765. doi: 10.3390/ijms241612765 (PMC10454525; doi:10.3390/ijms241612765)
Supplement: Supplementary file 1 [file ijms-24-12765-s001.zip › ijms-2507954-supplementary.pdf]

Supplementary Figure Legends

Supplementary Figure S1 Effects of perfluorooctane sulfonate (PFOS) on glucocorticoid receptor (GR)- or estrogen receptor (ER)-mediated transcription.

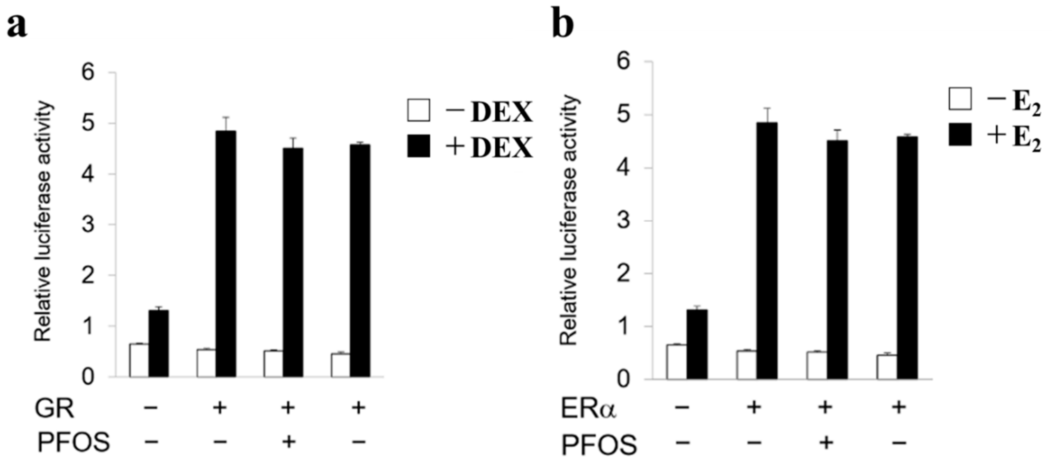

a,b : Expression plasmids encoding GR (*a;* 10 ng) or ERα (*b*; 10 ng) were co-transfected with mouse mammary tumor virus (MMTV)- luciferase (LUC) (a) or 2×estrogen response element (ERE) containing luciferase reporter (2xERE-LUC) (b) (100 ng) into CV-1 cells. Cells were incubated with or without 10^{-7} M dexamethasone (Dex) (a) or β-estradiol (E_2) (b) and the indicated amounts of PFOS. The total amount of DNA in each well was balanced by adding the vector pcDNA3. Data are presented as the mean ± SEM of experiments performed in triplicate. No significant differences were observed with PFOS addition.

Supplementary Figure S2 Effects of perfluorooctane sulfonate (PFOS) and/or thyroxine (T_4) on cell viability of C6 cells.

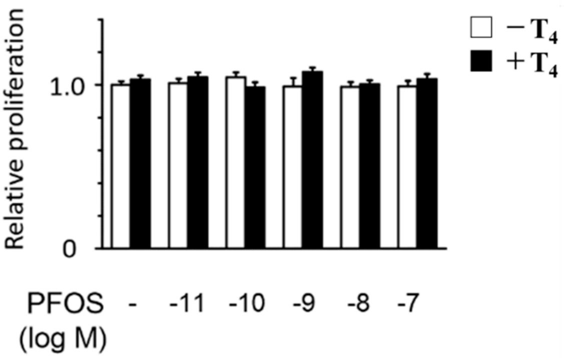

C6 cells in 96-well plates were incubated with or without the indicated concentrations of PFOS and/or T_4 for 24 h. Cell viability was determined via a proliferation assay using the CellTiter 96 AQueous One Solution Cell Proliferation Assay Kit (Promega) according to the manufacturer's instructions and indicated by relative proliferation as PFOS(-)=1.0. Values are means ± SEM of three experiments performed in triplicate. No significant differences were observed between groups.
